# Knowledge, attitudes, and practices regarding diabetic foot care among patients with diabetes in China: a systematic review and meta-analysis

**DOI:** 10.3389/fpubh.2026.1922404

**Published:** 2026-09-04

**Authors:** Yong Xiao, Shiyun Liu, Yueyue Wang, Fang Wang

**Affiliations:** 1 School of Nursing, Chengdu University of Traditional Chinese Medicine, Chengdu, Sichuan, China; 2 Dean’s Office, Guang’an Hospital of Traditional Chinese Medicine, Guang'an, Sichuan, China

**Keywords:** diabetes mellitus, diabetic foot, foot care, knowledge, attitudes, practice, meta-analysis

## Abstract

**Background:**

Diabetic foot is a serious and largely preventable complication of diabetes. Patient knowledge, attitudes, and practices (KAP) regarding foot care are central to early prevention, yet the overall distribution of KAP levels among patients with diabetes in China remains unclear. This systematic review and meta-analysis aimed to estimate the pooled proportions of different levels of diabetic foot care-related KAP among patients with diabetes in China.

**Methods:**

The review was registered in PROSPERO (CRD420261328954) and followed PRISMA 2020 reporting guidance. PubMed, Embase, Scopus, the Cochrane Library, Web of Science, CINAHL Complete, SinoMed, CNKI, Wanfang, and VIP were searched from inception to March 2, 2026. Cross-sectional studies reporting extractable diabetic foot-care KAP data among Chinese adults with diabetes were included. Study quality was assessed using the 11-item Agency for Healthcare Research and Quality checklist. Random-effects proportion meta-analyses used the Freeman-Tukey double arcsine transformation. Subgroup, sensitivity, and small-study effect analyses were conducted where data allowed.

**Results:**

Twenty-four cross-sectional studies involving 6,124 participants across 13 Chinese provinces or municipalities were included. Fourteen studies contributed to the meta-analysis of knowledge, five to attitudes, and seventeen to practices. The pooled proportions of poor, satisfactory, and good knowledge were 33% (95% CI: 19–48%), 43% (95% CI: 33–54%), and 26% (95% CI: 17–37%), respectively. For attitudes, 33% (95% CI: 10–62%) of participants had unfavorable attitudes and 67% (95% CI: 38–90%) had favorable attitudes. For practices, the pooled proportions of poor, satisfactory, and good foot care practices were 45% (95% CI: 35–55%), 45% (95% CI: 38–53%), and 10% (95% CI: 6–14%), respectively. Substantial heterogeneity was observed across most outcomes, and subgroup analyses did not identify statistically significant differences by study region, diabetic foot status, or publication period.

**Conclusion:**

Chinese patients with diabetes generally showed favorable attitudes toward diabetic foot care, but knowledge remained insufficient and good foot-care practices were uncommon. Because all included studies were cross-sectional, the pooled estimates primarily characterize the distribution of KAP levels and should be interpreted descriptively. When considered alongside existing guidelines and intervention evidence, these findings may inform the development of risk-stratified, behavior-oriented, and system-supported strategies for diabetic foot prevention.

**Systematic review registration:**

Publicly accessible record: https://www.crd.york.ac.uk/PROSPERO/view/CRD420261328954. PROSPERO registration: CRD420261328954.

## 1 Introduction

Diabetic foot is one of the most disabling and costly lower-extremity complications of diabetes. It may progress from peripheral neuropathy, peripheral artery disease, deformity, and unrecognized minor trauma to ulceration, infection, hospitalization, lower-limb amputation, and premature death, thereby imposing substantial clinical, psychological, and socioeconomic burdens on patients, families, and health systems (1). The global burden of diabetes is continuing to increase. Recent estimates from the International Diabetes Federation indicate that approximately 589 million adults worldwide were living with diabetes in 2024, with a further increase projected by 2050; China accounts for a particularly large share of this burden, with approximately 147.9 million adults living with diabetes and an estimated prevalence of 11.9% (2). A recent Chinese projection study further suggested that, under a natural scenario, the age-standardized prevalence of diabetes in China may increase substantially by 2050, with marked geographic variation across provinces (3).

The clinical consequences of diabetic foot are severe and often preventable. Diabetic foot ulcers are associated with infection, recurrence, functional decline, amputation, high medical expenditure, and excess mortality (4, 5). People with diabetes have a substantially higher risk of lower-extremity amputation than those without diabetes (6), and mortality after diabetic foot ulceration or diabetes-related amputation is comparable with that of many malignant diseases (4). These outcomes indicate that diabetic foot prevention should not be regarded as an optional component of diabetes care, but as a core element of long-term chronic disease management.

Evidence-based prevention relies on early identification of at-risk feet, regular professional foot assessment, appropriate footwear, treatment of pre-ulcerative lesions, prompt management of minor trauma, and daily self-care by patients (7, 8). International guidelines recommend structured education and regular reminders for people at risk of foot ulceration, including daily inspection of the feet and footwear, avoidance of barefoot walking, appropriate skin and nail care, and timely contact with health professionals when new lesions are suspected (8, 9). Systematic reviews also suggest that educational interventions can improve diabetes-related foot care knowledge and behaviors, although knowledge transmission alone may be insufficient when behavioral capability, motivation, and follow-up support are weak (10, 11).

In China, the expanding diabetes population, regional disparities in health resources, and uneven access to specialist diabetic foot services make patient self-care particularly important. Nevertheless, routine foot self-care remains difficult for many patients. Chinese studies have reported gaps in daily foot inspection, appropriate foot hygiene, footwear selection, early help-seeking for foot problems, and regular professional assessment (12, 13). The knowledge, attitude and practice (KAP) framework provides a useful lens for describing these gaps, as knowledge may contribute to risk perception and attitudes, while favorable attitudes may support the adoption of protective behaviors. However, this relationship should not be interpreted as strictly linear. Behavioral skills, self-efficacy, health literacy, family support, accessibility of care, and repeated professional reinforcement may all influence whether knowledge and attitudes are translated into sustained daily practice (14, 15).

Several cross-sectional studies have examined KAP regarding diabetic foot care among patients with diabetes in China, but their estimates vary substantially because of differences in sample size, geographic region, study population, diabetic foot risk status, and assessment instruments. Consequently, the existing evidence remains fragmented, and the overall distribution of diabetic foot care-related KAP and its variation across key study characteristics remain unclear. To address this evidence gap, this systematic review and meta-analysis aimed to estimate the pooled proportions of poor, satisfactory, and good knowledge; favorable and unfavorable attitudes; and poor, satisfactory, and good practices regarding diabetic foot care among Chinese patients with diabetes. We also explored subgroup differences by study region, diabetic foot status, and publication period to identify priority gaps and inform targeted, structured, and behavior-oriented foot care interventions.

## 2 Methods

This review was registered in PROSPERO (CRD420261328954) and was reported in accordance with the Preferred Reporting Items for Systematic Reviews and Meta-Analyses (PRISMA) 2020 statement (16).

### 2.1 Search strategy

Two reviewers (YX and YW) independently searched PubMed, Embase, Scopus, the Cochrane Library, Web of Science, CINAHL Complete, SinoMed,^1^ CNKI,^2^ Wanfang,^3^ and VIP^4^ from database inception to March 2, 2026. The search strategy combined controlled vocabulary and free-text terms for three concepts: diabetic foot or foot care; self-care, self-management, or preventive care; and knowledge, attitude, practice, behavior, or KAP. Boolean operators were adapted for each database. Reference lists of eligible studies were also screened to identify additional studies. The complete database-specific strategies are provided in Supplementary Table S1. A supplementary search of medRxiv, Research Square, and ChinaXiv identified no additional potentially eligible preprints.

### 2.2 Inclusion criteria

Studies were considered eligible when they satisfied the following criteria: (1) full-text reports of primary research, including journal articles and academic theses/dissertations, with sufficient information for eligibility assessment and data extraction; (2) Chinese or English language; (3) cross-sectional design; (4) participants were adults aged 18 years or older with diabetes in China, with or without diabetic foot ulcers; and (5) the study reported extractable percentage or count data for at least one KAP domain related to diabetic foot care or diabetic foot prevention.

### 2.3 Exclusion criteria

Studies were excluded if they (1) were conducted in non-Chinese populations; (2) did not report relevant KAP outcomes or did not provide data that could be converted into proportions; (3) were conference abstracts or proceedings, unpublished reports without sufficient full-text data, protocols, reviews, commentaries, editorials, case reports, or other secondary research; (4) were duplicate publications using the same dataset, in which case the most complete report was retained; or (5) were published in languages other than Chinese or English.

### 2.4 Methodological quality assessment

The methodological quality of included cross-sectional studies was assessed using the 11-item checklist recommended by the Agency for Healthcare Research and Quality (AHRQ) for cross-sectional studies (17). Each item was scored as 1 for ‘yes’ and 0 for ‘no’ or ‘unclear’, yielding total scores from 0 to 11. Studies were classified as low quality (0–3), moderate quality (4–7), or high quality (8–11). Two reviewers (YX and YW) independently assessed study quality, and disagreements were resolved through discussion with a third reviewer (SL).

### 2.5 Study selection

Retrieved records were imported into EndNote for de-duplication and management. Two reviewers (YX and YW) independently screened titles and abstracts against the eligibility criteria and then assessed potentially eligible full texts. Two reviewers independently assessed the full-text articles for eligibility. Any disagreements were resolved through discussion and consultation with a third reviewer (SL), after which studies meeting all predefined criteria were included.

### 2.6 Data extraction

Two reviewers independently extracted data using a standardized data extraction form informed by the Joanna Briggs Institute framework for systematic reviews of prevalence evidence. Extracted information included first author, publication year, study design, study region, sample size, population characteristics, diabetic foot status, assessment instrument, instrument validation status, and the numbers or proportions of participants in each KAP category. Because the included studies used different assessment instruments, scoring ranges, and classification thresholds, no common numerical cut-off was imposed. Instead, the definitions and criteria reported in the original studies were retained, and the corresponding outcomes were harmonized into common KAP categories: poor, satisfactory, and good for knowledge and practice, and unfavorable and favorable for attitude. When only percentages were reported, counts were calculated using the reported sample size. When multiple relevant indicators were available, the indicator most directly related to diabetic foot care KAP was prioritized. Discrepancies were resolved by discussion or consultation with a third reviewer.

### 2.7 Statistical analysis

All statistical analyses were conducted in R (version 4.5.3) using the ‘meta’ package (version 6.0.0) and the ‘metafor’ package (version 3.8.1). Meta-analyses of proportions were performed to estimate pooled proportions for each KAP category. Between-study heterogeneity was assessed using Cochran’s *Q* test and the *I*^2^ statistic, with *I*^2^ values of <25%, 25–50%, and >50% interpreted as low, moderate, and substantial heterogeneity, respectively. Because clinical and methodological heterogeneity was expected across cross-sectional surveys, random-effects models were used. The Freeman–Tukey double arcsine transformation was selected to stabilize variances in the meta-analysis of proportions, particularly when study-level proportions were very low or very high; pooled estimates were subsequently back-transformed for interpretation. For outcomes with substantial heterogeneity and sufficient available data, subgroup analyses were conducted using predefined study-level variables, including study region, diabetic foot status, and publication period. These variables were selected *a priori* to explore potentially relevant geographic, clinical, and temporal sources of between-study variability. Leave-one-out sensitivity analyses were performed by sequentially excluding one study at a time and recalculating the pooled estimate. Small-study effects or publication bias were evaluated when at least 10 studies were available for an outcome, using funnel plots and Egger’s regression test. Because trim-and-fill estimates can be unstable in the presence of high heterogeneity, trim-and-fill analyses were interpreted as exploratory.

## 3 Results

### 3.1 Literature screening for systematic analysis

**Figure 1:**
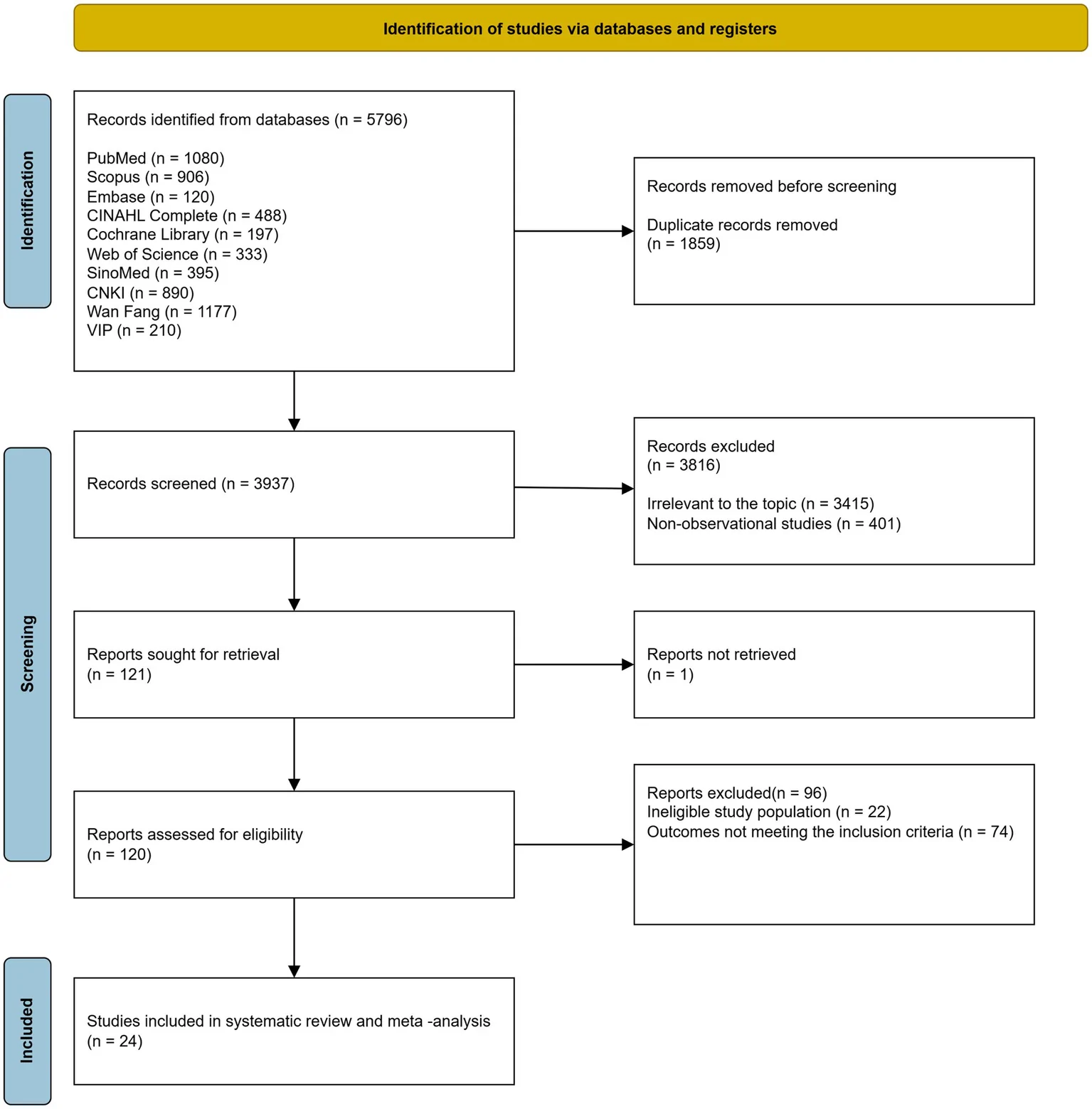
Preferred reporting items for systematic review and meta-analysis (PRISMA) flow diagram.

The study selection process is shown in Figure 1. A total of 5,796 records were identified through comprehensive database searching, including PubMed (*n* = 1,080), Scopus (*n* = 906), Embase (*n* = 120), CINAHL Complete (*n* = 488), Cochrane Library (*n* = 197), Web of Science (*n* = 333), SinoMed (*n* = 395), CNKI (*n* = 890), Wanfang (*n* = 1,177), and VIP database (*n* = 210). After removing 1,859 duplicate records, 3,937 records remained for title and abstract screening. Of these, 3,816 records were excluded, including 3,415 records unrelated to the topic and 401 non-observational studies. A total of 121 reports were sought for retrieval, of which one report could not be retrieved. Therefore, 120 reports were assessed for eligibility. Among these, 96 reports were excluded because of ineligible study populations (*n* = 22) or outcomes not meeting the inclusion criteria (*n* = 74). Finally, 24 studies were included in the systematic review, with eligible data synthesized quantitatively by outcome (18–41).

### 3.2 Studies included in each meta-analysis

The studies included in the systematic review were not entirely identical to those included in each meta-analysis. Although 24 cross-sectional studies were included in the systematic review, not all studies provided extractable data for quantitative synthesis across all three KAP domains because of differences in the outcomes reported and classification methods used. Consequently, 14 studies contributed to the meta-analysis of knowledge, 5 to attitudes, and 17 to practices. The exact number of studies included for each outcome is presented in the corresponding forest plots.

### 3.3 Study characteristics

Overall, 24 cross-sectional studies were included, representing 6,124 participants with diabetes from 13 provinces or municipalities in China. The largest number of studies was conducted in Guangdong Province (*n* = 6), followed by Beijing Municipality (*n* = 4). All included studies used a cross-sectional design. Most studies focused on patients with type 2 diabetes mellitus; six studies included patients with diabetic foot ulcers, and four studies included patients at high risk of diabetic foot. Regarding questionnaire validation, six studies used previously validated questionnaires and additionally assessed their reliability or validity in the study samples; 15 studies adopted previously validated questionnaires without further validation; and three studies used unvalidated structured questionnaires. Further characteristics of the included studies are summarized in Table 1.

**Table 1:** Basic characteristics and quality evaluation of included literature.

First author	Region	Population	Sample size	Assessment instrument	Distribution across KAP dimensions, *n*								Quality score
					Poor knowledge	Satisfactory knowledge	Good knowledge	Unfavorable attitude	Favorable attitude	Poor practices	Satisfactory practices	Good practices	
Lianggui et al. (2010) (18)	Hunan	Patients with type 2 diabetes	102	Validated structured questionnaire	NR	NR	NR	53	49	19	58	25	6
Enhe et al. (2011) (19)	Anhui	Patients with diabetes	180	Unvalidated structured questionnaire	63	85	32	NR	NR	NR	NR	NR	4
Ting et al. (2011) (20)	Guangdong	Patients with diabetes	110	Unvalidated structured questionnaire	78	32	NR	NR	NR	93	17	NR	5
Hongju et al. (2013) (21)	Hubei	Patients with type 2 diabetes	150	Previously validated questionnaire	NR	NR	NR	NR	NR	36	81	33	6
Li et al. (2015) (22)	Tianjin	Patients with type 2 diabetes	300	Previously validated questionnaire	NR	NR	NR	NR	NR	157	143	NR	8
Liping et al. (2016) (23)	Beijing	Patients with type 2 diabetes	178	Unvalidated structured questionnaire	34	125	19	NR	NR	53	115	10	5
Enfang et al. (2017) (24)	Shanghai	Patients with type 2 diabetes at high risk of diabetic foot	179	Validated structured questionnaire	148	31	NR	NR	NR	NR	NR	NR	7
Liangqin et al. (2019) (25)	Guangdong	Patients with diabetes	958	Previously validated questionnaire	37	576	345	NR	NR	NR	NR	NR	9
Xu et al. (2019) (26)	Shanghai	Patients with type 2 diabetes	106	Previously validated questionnaire	31	75	NR	NR	NR	54	52	NR	8
Liu et al. (2020) (27)	Qingdao	Patients with diabetic foot	203	Previously validated questionnaire	62	64	77	NR	NR	NR	NR	NR	7
Miaomiao (2020) (28)	Xi’an	Patients with type 2 diabetes without diabetic foot	200	Validated structured questionnaire	170	20	10	NR	NR	NR	NR	NR	9
Yang et al. (2020) (29)	Beijing	Patients with type 2 diabetes	140	Previously validated questionnaire	NR	NR	NR	NR	NR	67	65	8	6
Ting (2021) (32)	Guangdong	Patients with type 2 diabetes and diabetic foot	95	Previously validated questionnaire	16	59	20	21	74	17	63	15	6
Long et al. (2021) (30)	Sichuan	Patients with type 2 diabetes	204	Previously validated questionnaire	8	86	110	NR	NR	NR	NR	NR	8
Qin et al. (2021) (31)	Anhui	Patients with type 2 diabetes complicated with DF	90	Previously validated questionnaire	27	29	34	NR	NR	NR	NR	NR	7
Yang et al. (2021) (33)	Guangdong	Patients with type 2 diabetes complicated by DF	343	Previously validated questionnaire	NR	NR	NR	246	97	176	136	31	7
Lin et al. (2022) (35)	Shanghai	Patients with diabetes	411	Validated structured questionnaire	NR	NR	NR	NR	NR	238	112	61	7
Jia et al. (2022) (34)	Shanxi	Patients with diabetes	1,080	Previously validated questionnaire	271	557	252	390	690	771	276	33	8
Qiuping et al. (2023) (38)	Beijing	Older patients with diabetes at high risk of diabetic foot	210	Previously validated questionnaire	NR	NR	NR	NR	NR	126	80	4	8
Na (2023) (37)	Henan	Older patients with type 2 diabetes at high risk of diabetic foot	305	Previously validated questionnaire	108	156	41	NR	NR	116	153	36	8
Chenxia et al. (2023) (36)	Guangdong	Patients with type 2 diabetes	156	Validated structured questionnaire	NR	NR	NR	NR	NR	104	47	5	6
Yi-Jun et al. (2023) (39)	Zhejiang	Patients with type 2 diabetes at high risk of diabetic foot	107	Previously validated questionnaire	NR	NR	NR	NR	NR	45	55	7	6
Xiaomei et al. (2025) (41)	Guangdong	Patients with type 2 diabetes and diabetic foot	111	Previously validated questionnaire	NR	NR	NR	NR	NR	36	60	14	4
Qi et al. (2025) (40)	Beijing	Patients with type 2 diabetes	206	Validated structured questionnaire	29	75	100	3	203	37	129	40	8

NR, not reported in the original study.

### 3.4 Methodological quality of the included studies

The quality appraisal is summarized in Supplementary Table S2. Of the 24 studies included, 9 were classified as high quality and 15 as moderate quality. Overall, most included studies had moderate-to-high methodological quality, yet some studies had limitations such as convenience sampling, inadequate reporting of missing data, lack of blinding for assessors, and insufficient control of potential confounding factors.

### 3.5 Meta-analysis

#### 3.5.1 Pooled knowledge regarding diabetic foot care

**Figure 2:**
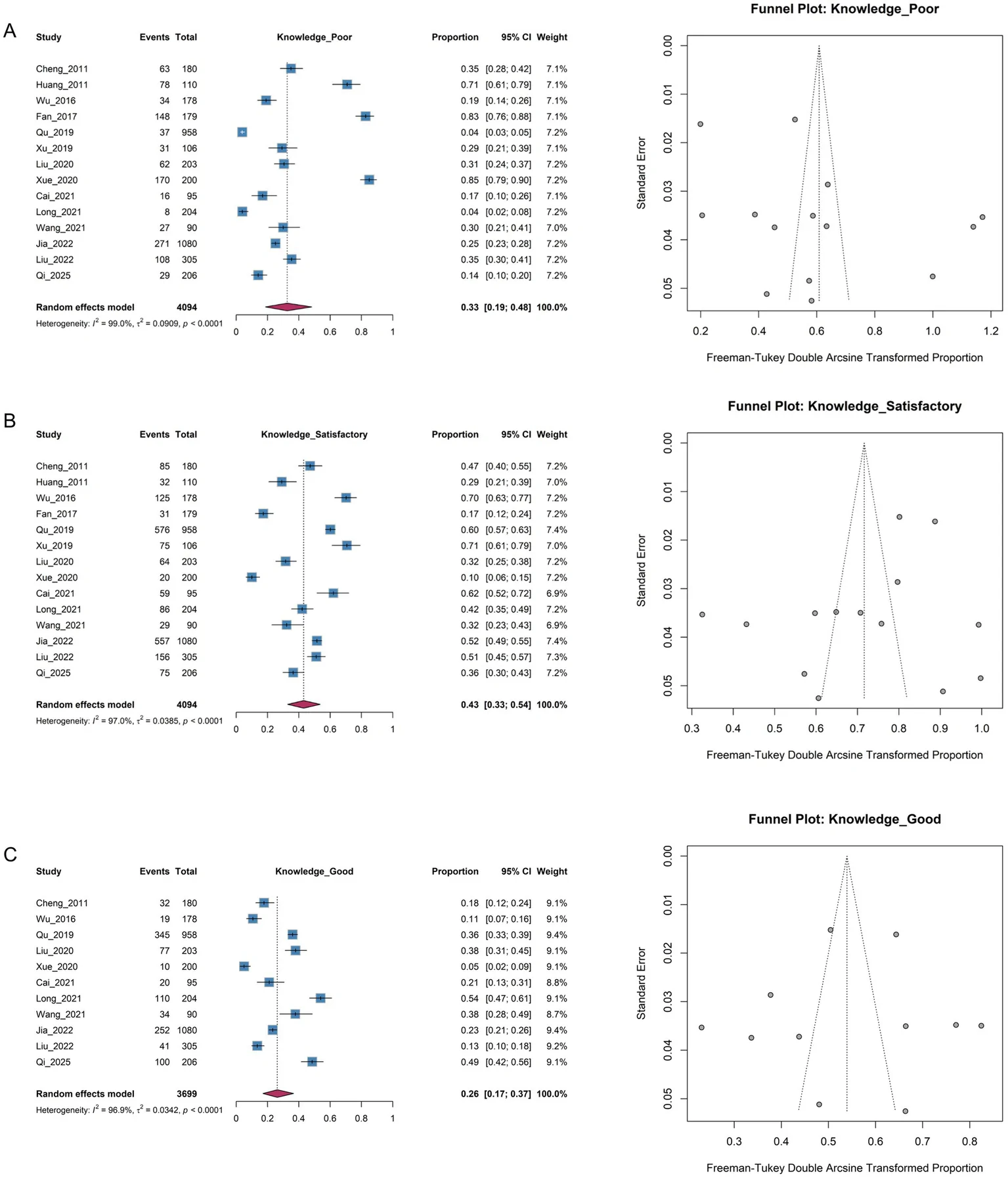
Forest plots and funnel plots of diabetic foot care knowledge among Chinese patients with diabetes **(A)** poor knowledge; **(B)** satisfactory knowledge; and **(C)** good knowledge.

Fourteen studies involving 4,094 participants reported the proportion of poor knowledge regarding diabetic foot care. The random-effects pooled proportion was 33% (95% CI: 19–48%), with substantial heterogeneity across studies (*I*^2^ = 99.0%, *p* < 0.001). Egger’s test showed no evidence of significant small-study effects (*t* = 1.78, df = 12, *p* = 0.100; Figure 2A).

For satisfactory knowledge, 14 studies involving 4,094 participants were included. The pooled proportion was 43% (95% CI: 33–54%), and substantial heterogeneity was observed (*I*^2^ = 97.0%, *p* < 0.001). Egger’s test did not indicate significant funnel plot asymmetry (*t* = −1.51, df = 12, *p* = 0.156; Figure 2B).

For good knowledge, 11 studies involving 3,699 participants were included. The pooled proportion was 26% (95% CI: 17–37%), with substantial heterogeneity among studies (*I*^2^ = 96.9%, *p* < 0.001). Egger’s test showed no evidence of significant small-study effects (*t* = −0.27, df = 9, *p* = 0.794; Figure 2C).

#### 3.5.2 Pooled attitudes toward diabetic foot care

**Figure 3:**
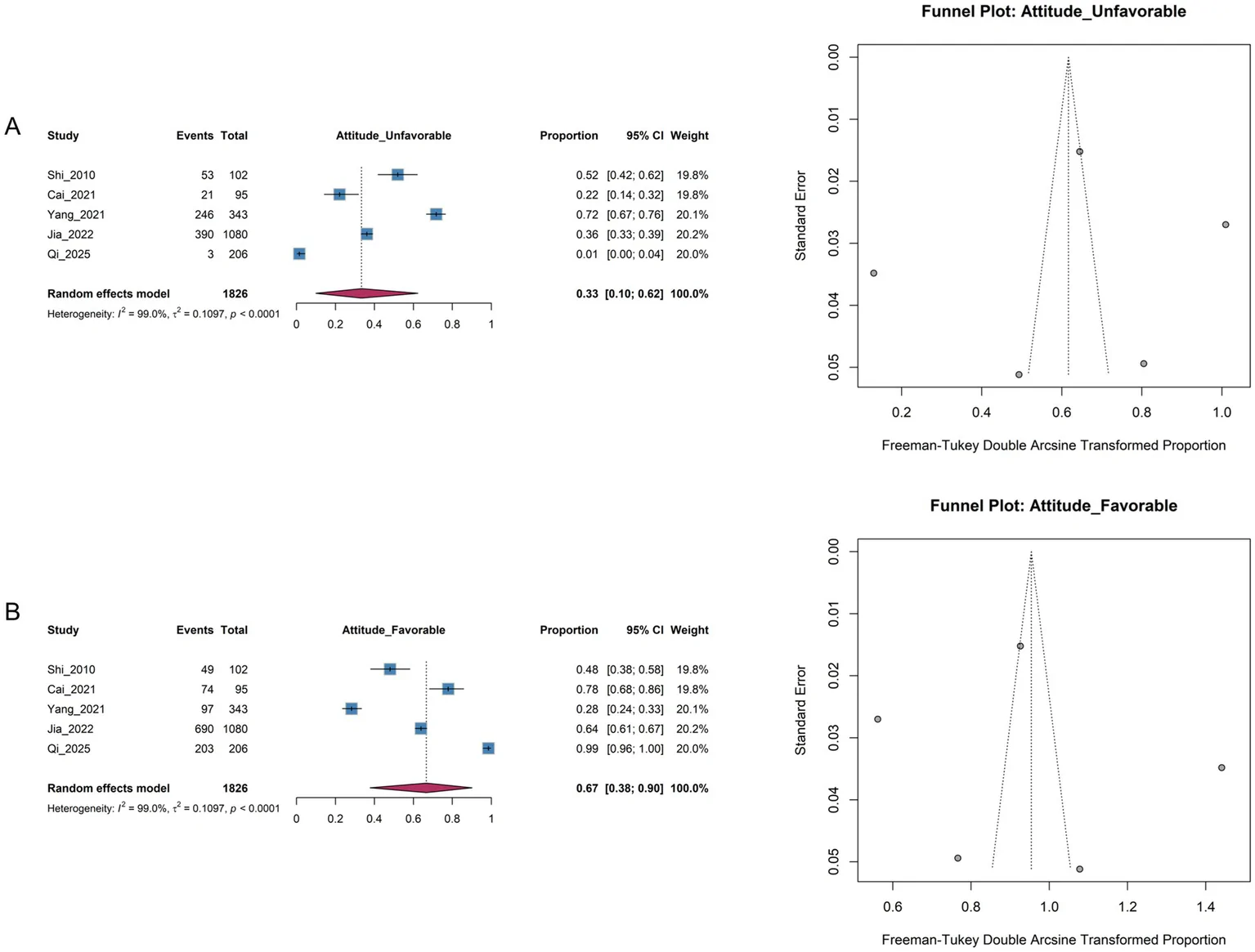
Forest plots and funnel plots of attitudes toward diabetic foot care among Chinese patients with diabetes: **(A)** Unfavorable attitudes; and **(B)** Favorable attitudes.

Five studies involving 1,826 participants were included in the meta-analysis of attitudes toward diabetic foot care. The pooled proportion of participants with unfavorable attitudes was 33% (95% CI: 10–62%; Figure 3A), whereas the pooled proportion with favorable attitudes was 67% (95% CI: 38–90%; Figure 3B). Substantial heterogeneity was observed for both outcomes (*I*^2^ = 99.0%, *p* < 0.001). Because fewer than 10 studies were available, small-study effects were not formally assessed for the attitude outcomes; the funnel plots in Figure 3 are shown only for descriptive visual reference and were not used to infer funnel plot asymmetry or publication bias.

#### 3.5.3 Pooled practices regarding diabetic foot care

**Figure 4:**
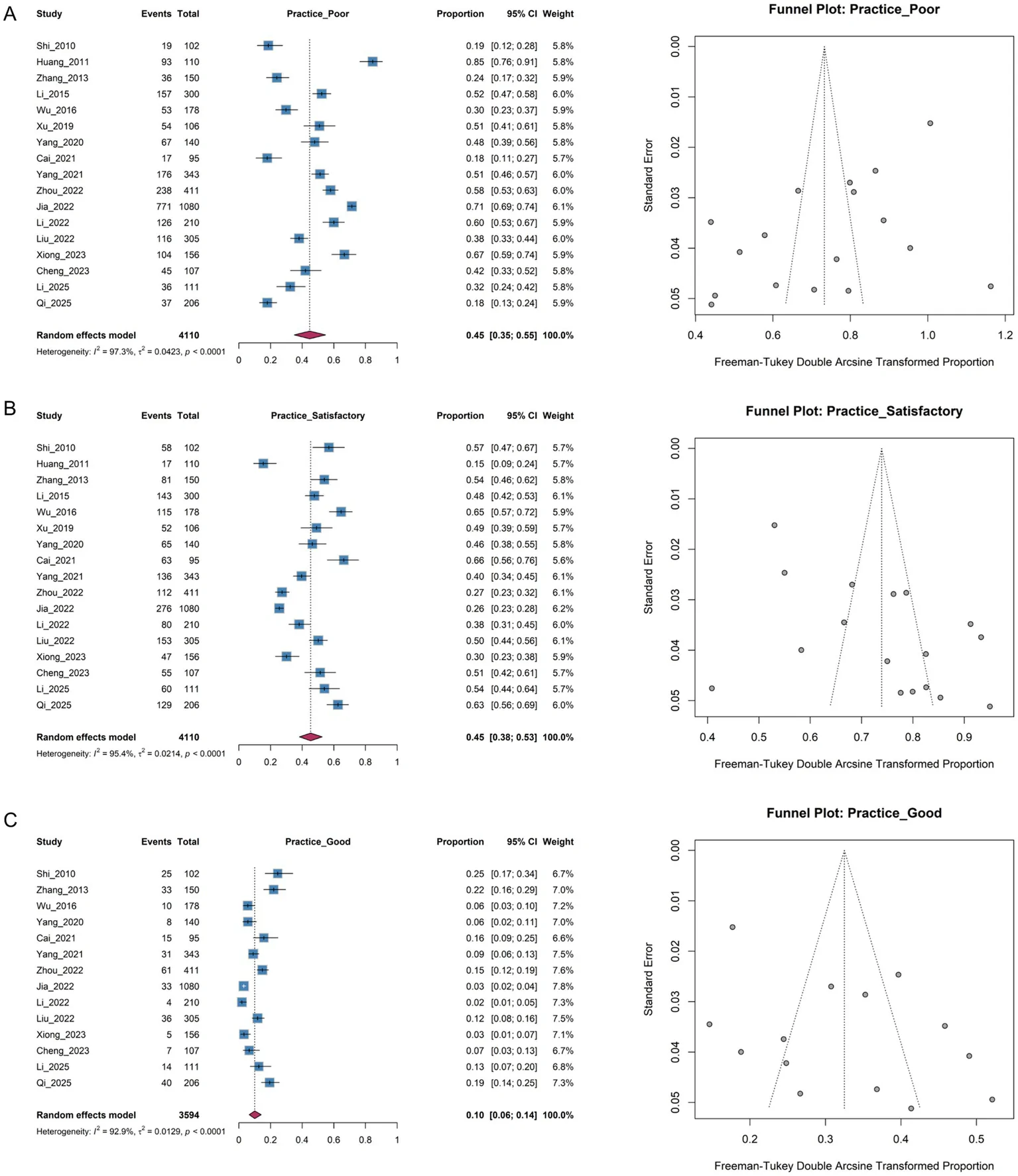
Forest plots and funnel plots of diabetic foot care practices among Chinese patients with diabetes **(A)** poor practices; **(B)** satisfactory practices; and **(C)** good practices.

Seventeen studies involving 4,110 participants reported data on poor foot care practices. The pooled proportion of participants with poor practices was 45% (95% CI: 35–55%; Figure 4A), with substantial heterogeneity across studies (*I*^2^ = 97.3%, *p* < 0.001). Egger’s test indicated significant funnel plot asymmetry, suggesting potential small-study effects (*t* = −2.97, df = 15, *p* = 0.009).

For satisfactory practices, 17 studies involving 4,110 participants were included. The pooled proportion with satisfactory practices was 45% (95% CI: 38–53%; Figure 4B), with substantial heterogeneity across studies (*I*^2^ = 95.4%, *p* < 0.001). Egger’s linear regression test indicated significant funnel plot asymmetry (*t* = 3.22, df = 15, *p* = 0.006), suggesting potential small-study effects or publication bias.

For good practices, 14 studies involving 3,594 participants were included. The pooled proportion of participants with good practices was 10% (95% CI: 6–14%; Figure 4C), with substantial heterogeneity among studies (*I*^2^ = 92.9%, *p* < 0.001). Egger’s test did not indicate statistically significant funnel plot asymmetry (*t* = 2.08, df = 12, *p* = 0.059).

### 3.6 Subgroup analyses of knowledge and practices regarding diabetic foot care

Subgroup analyses were performed by study region, diabetic foot status, and publication period to explore potential sources of heterogeneity in poor knowledge and poor practice outcomes. Heterogeneity remained substantial within most subgroups, with *I*^2^ values generally exceeding 90%, indicating that the predefined subgroup variables did not fully account for the observed between-study heterogeneity. The persistently high residual heterogeneity indicates that the exact magnitude of the pooled proportions should be interpreted with caution. Subgroup analyses were not conducted for attitude outcomes because of the limited number of available studies.

For poor knowledge, no significant subgroup differences were observed by study region (31.2% in southern China vs. 36.3% in northern China; *p* for subgroup difference = 0.771) or diabetic foot status (*p* for subgroup difference = 0.449). Studies published after 2020 showed a lower pooled proportion of poor knowledge than those published in 2020 or earlier (19.6% vs. 43.3%), but the difference was not statistically significant (*p* for subgroup difference = 0.063).

For poor practices, no significant subgroup differences were found by study region (44.5% in southern China vs. 40.5% in northern China; *p* for subgroup difference = 0.687), diabetic foot status (*p* for subgroup difference = 0.987), or publication period (43.9% in 2020 or earlier vs. 45.3% after 2020; *p* for subgroup difference = 0.895). Detailed results are shown in Table 2.

**Table 2:** Subgroup analyses of poor knowledge and poor practices regarding diabetic foot care.

Outcome	Subgroup variable	Subgroup	No. of studies	Heterogeneity *p* value	*I* ^2^	Pooled proportion (95% CI)	*p*-Value
Poor knowledge	Study region	South	8	<0.001	99.1%	0.312 (0.121–0.544)	0.771
		North	5	<0.001	98.7%	0.363 (0.133–0.632)	
	Diabetic foot status	Without diabetic foot	9	<0.001	99.3%	0.390 (0.186–0.616)	0.449
		At high risk of diabetic foot	2	<0.001	98.9%	0.166 (0.000–0.561)	
		With diabetic foot	3	0.028	72.1%	0.258 (0.175–0.351)	
	Publication period	2020 or earlier	8	<0.001	99.4%	0.433 (0.209–0.672)	0.063
		After 2020	6	<0.001	95.5%	0.196 (0.106–0.305)	
Poor practices	Study region	South	10	<0.001	96.3%	0.445 (0.305–0.589)	0.687
		North	6	<0.001	95.6%	0.405 (0.281–0.536)	
	Diabetic foot status	Without diabetic foot	10	<0.001	98.1%	0.453 (0.308–0.603)	0.987
		At high risk of diabetic foot	3	0.003	83.1%	0.440 (0.357–0.524)	
		With diabetic foot	4	<0.001	96.5%	0.438 (0.219–0.671)	
	Publication period	2020 or earlier	7	<0.001	96.5%	0.439 (0.269–0.616)	0.895
		After 2020	10	<0.001	97.7%	0.453 (0.332–0.578)	

### 3.7 Sensitivity analysis

To examine whether the pooled estimates were driven by any single study, we conducted leave-one-out analyses for the knowledge, attitude, and practice outcomes. Each study was sequentially excluded, and the pooled proportions were recalculated using a random-effects model. The recalculated estimates and 95% CIs were broadly consistent with the primary analyses, indicating that no individual study materially changed the overall results. The detailed leave-one-out results are presented in Supplementary Table S3; Supplementary Figures S1–S8.

### 3.8 Publication bias

Egger’s linear regression test was used to assess funnel plot asymmetry for outcomes with at least 10 included studies. No statistically significant evidence of funnel plot asymmetry was observed for poor knowledge (*t* = 1.78, *p* = 0.100), satisfactory knowledge (*t* = −1.51, *p* = 0.156), or good knowledge (*t* = −0.27, *p* = 0.794). For good practices, Egger’s test did not reach statistical significance but showed a borderline result (*t* = 2.08, *p* = 0.059), suggesting that potential small-study effects could not be completely excluded. Significant funnel plot asymmetry was detected for poor practices (*t* = −2.97, *p* = 0.009) and satisfactory practices (*t* = 3.22, *p* = 0.006), indicating potential small-study effects or publication bias. Formal assessment of small-study effects was not conducted for attitude outcomes because fewer than 10 studies were available, and the attitude funnel plots were presented only as descriptive visual displays.

For practice outcomes showing significant or borderline funnel plot asymmetry, trim-and-fill analyses were further conducted as exploratory sensitivity analyses. For poor practices, six potentially missing studies were imputed, and the adjusted pooled proportion was 58.5% (95% CI: 46.2–70.3%). For satisfactory practices, six studies were imputed, yielding an adjusted pooled proportion of 36.0% (95% CI: 27.9–44.5%). For good practices, five studies were imputed in an exploratory analysis, and the adjusted pooled proportion was 5.5% (95% CI: 2.4–9.8%). Although the adjusted proportions differed from the primary estimates, the overall pattern remained unchanged, with good foot-care practices remaining uncommon and poor or satisfactory practices accounting for the majority of participants. However, substantial heterogeneity persisted after trim-and-fill adjustment; therefore, these adjusted estimates were interpreted as exploratory and did not replace the primary random-effects estimates. The corresponding trim-and-fill funnel plots are presented in Supplementary Figures S9–S11.

## 4 Discussion

This systematic review and meta-analysis synthesized evidence on knowledge, attitudes, and practices regarding diabetic foot care among patients with diabetes in China. The main finding was a clear imbalance across the KAP domains: attitudes were relatively favorable, whereas knowledge was insufficient and actual foot care practices were poor. Specifically, the pooled proportions of poor, satisfactory, and good knowledge were 33, 43, and 26%, respectively; 67% of participants had favorable attitudes; and only 10% demonstrated good foot care practices. These findings indicate that diabetic foot prevention in China faces not only an information gap, but also a substantial implementation gap between recognizing the importance of foot care and performing protective behaviors consistently.

The low proportion of good knowledge suggests that many patients may not fully understand the mechanisms, warning signs, and preventive actions related to diabetic foot. This finding is consistent with Chinese KAP studies showing that patients often have incomplete knowledge of foot examination, early treatment of foot problems, and risk factors for ulceration (15, 34). A large survey of rural adults with diabetes in northern China similarly reported suboptimal diabetic foot prevention knowledge, particularly in items related to professional foot examination and the management of foot problems (34). The present subgroup analysis suggested that studies published after 2020 had a lower pooled proportion of poor knowledge than earlier studies, although the difference was not statistically significant. This may reflect increasing attention to chronic disease management, national diabetic foot guidelines, and community diabetes services in China (42, 43). However, the persistence of poor or only satisfactory knowledge may partly reflect gaps in the delivery, reinforcement, and tailoring of foot-care education.

A more clinically important finding is the discrepancy between favorable attitudes and poor practices. Although two-thirds of patients had favorable attitudes, only one in ten achieved good foot care practices. This pattern is consistent with individual studies in which patients expressed positive attitudes but still reported poor daily foot inspection, inadequate footwear behaviors, and delayed help-seeking (14). This finding supports caution against assuming a simple linear pathway from knowledge and attitudes to practice. Positive attitudes may reflect general approval of foot care, but daily foot self-care is a repetitive preventive behavior that often produces few immediate or visible benefits. Therefore, behaviors such as inspecting the soles, checking inside shoes, avoiding barefoot walking, moisturizing dry skin appropriately, seeking professional help for calluses or wounds, and attending regular foot assessments may be difficult to sustain unless patients have sufficient behavioral skills, self-efficacy, cues to action, and accessible professional support (44–46).

The observed practice gap is clinically relevant to ulcer prevention and may inform the design of future interventions. Because diabetic peripheral neuropathy can reduce pain perception, minor trauma may remain unnoticed until ulceration or infection develops. Poor daily inspection, inappropriate footwear, and delayed treatment of minor lesions may therefore accelerate the pathway from preventable trauma to ulceration and amputation. Current international and Chinese guidance emphasizes structured education, regular risk-based foot screening, appropriate footwear, treatment of pre-ulcerative lesions, and integrated care for moderate- and high-risk patients (8, 43). When interpreted alongside these guidelines and broader intervention evidence, our findings suggest that future diabetic foot prevention strategies may need to move beyond one-time, knowledge-based education toward more behavior-oriented and system-supported approaches. Evidence-informed strategies may include demonstration and return demonstration of foot-care skills, individualized education tailored to foot-risk status and personal circumstances, written and visual materials appropriate to patients’ health literacy, family involvement where appropriate, and repeated reinforcement during routine diabetes follow-up (9, 47). Such education should be embedded in primary care, community health centers, endocrinology clinics, and wound care services, with content stratified by risk level (9, 48). Patients without foot complications need basic prevention and annual screening, whereas those at high risk or with previous ulceration require more frequent review, footwear assessment, rapid referral pathways, and multidisciplinary support. Digital health strategies may complement face-to-face education, as recent evidence suggests that mobile health interventions can improve foot care behaviors, knowledge, and beliefs, and nurse-led telehealth foot care programs appear feasible and acceptable (49, 50). Nevertheless, digital interventions should be designed with attention to older adults, rural patients, and patients with limited digital literacy; otherwise, they may widen existing disparities.

The non-significant subgroup findings should be interpreted cautiously. Poor knowledge and poor practices did not differ significantly by study region, diabetic foot status, or publication period, and heterogeneity remained substantial within most subgroups. This suggests that gaps in diabetic foot care knowledge and practices may not be confined to a particular geographic area or patient subgroup. However, the lack of statistically significant subgroup differences does not prove equivalence. The subgroup analyses were based on study-level rather than individual-level data and may have been underpowered, particularly for diabetic foot status categories. In addition, the substantial residual heterogeneity indicates that the predefined study-level variables did not fully account for the observed between-study variability. The residual heterogeneity may reflect between-study differences beyond the predefined subgroup dimensions, including differences in KAP assessment instruments, scoring methods and classification cut-offs, as well as participants’ socioeconomic characteristics. Variations in study settings, prior exposure to diabetes education, health literacy, and access to foot-care services may also have contributed to the observed heterogeneity. Accordingly, the substantial between-study heterogeneity warrants caution when interpreting the exact magnitude of the pooled proportions. Nevertheless, the pooled estimates remained broadly stable in the leave-one-out sensitivity analyses, indicating that the overall pattern of findings was not materially altered by the exclusion of any single study.

This review has several strengths and limitations. Strengths include a comprehensive search of both international and Chinese databases, inclusion of studies from multiple regions of China, quantitative synthesis of KAP levels, methodological quality assessment, subgroup and sensitivity analyses, and assessment of small-study effects where data allowed. Several limitations should also be noted. First, all included studies were cross-sectional, which precludes causal inference between knowledge, attitudes, and practices. Second, assessment instruments, scoring rules, and classification criteria varied substantially across studies, contributing to high measurement heterogeneity that was not fully explained by predefined subgroup analyses. Third, attitude outcomes were based on only five studies, limiting the precision of the estimates; future reviews may integrate qualitative evidence to provide a more nuanced understanding of patients’ attitudes. Fourth, self-reported practices may be affected by recall and social desirability biases, and future studies could incorporate objective measures, such as professional observation of foot-inspection techniques or clinical documentation of footwear choices. Finally, meta-regression was not performed because of the limited number of studies for each outcome and uneven reporting of study-level covariates. Future studies should use validated instruments with transparent scoring rules, report both domain-level scores and key item-level behaviors, and adopt longitudinal or intervention designs to determine whether improvements in knowledge and attitudes translate into sustained foot-care behaviors and reduced ulceration risk.

## 5 Conclusion

In conclusion, Chinese patients with diabetes generally show favorable attitudes toward diabetic foot care, but knowledge remains insufficient and good foot-care practices are uncommon. Given the cross-sectional nature of the included evidence, these pooled estimates should be interpreted as describing the distribution of KAP levels. The findings may inform the development of standardized, risk-stratified, behavior-oriented, and system-supported strategies for diabetic foot prevention.

## Data Availability

The original contributions presented in the study are included in the article/Supplementary material, further inquiries can be directed to the corresponding author/s.

## Footnotes

1
https://www.sinomed.ac.cn/zh/

2
https://oversea.cnki.net/

3
https://www.wanfangdata.com.cn/

4
https://www.cqvip.com/
