# Supplementary material for: Knowledge, attitudes, and practices regarding diabetic foot care among patients with diabetes in China: a systematic review and meta-analysis
Source: Front Public Health. 2026 Sep 4;14:1922404. doi: 10.3389/fpubh.2026.1922404 (PMC13586011; doi:10.3389/fpubh.2026.1922404)
Supplement: Supplementary file 1 [file Data_Sheet_1.docx]

**Supplementary Table S1** Search strategies in each included database

| PubMed | | |
| --- | --- | --- |
| 3/2/2026 | | |
| #1 | diabetic foot[MeSH Terms] | 13,710 |
| #2 | (("diabete"[All Fields] OR "diabetes mellitus"[MeSH Terms] OR ("diabetes"[All Fields] AND "mellitus"[All Fields]) OR "diabetes mellitus"[All Fields] OR "diabetes"[All Fields] OR "diabetes insipidus"[MeSH Terms] OR ("diabetes"[All Fields] AND "insipidus"[All Fields]) OR "diabetes insipidus"[All Fields] OR "diabetic"[All Fields] OR "diabetics"[All Fields] OR "diabets"[All Fields]) AND ("foot"[MeSH Terms] OR "foot"[All Fields])) OR ("diabetic foot"[MeSH Terms] OR ("diabetic"[All Fields] AND "foot"[All Fields]) OR "diabetic foot"[All Fields]) OR ("diabetic foot"[MeSH Terms] OR ("diabetic"[All Fields] AND "foot"[All Fields]) OR "diabetic foot"[All Fields] OR ("diabetic"[All Fields] AND "foot"[All Fields] AND "ulcer"[All Fields]) OR "diabetic foot ulcer"[All Fields] OR "DFU"[All Fields] OR (("foot"[MeSH Terms] OR "foot"[All Fields]) AND "ulcer*"[All Fields])) | 33,011 |
| #3 | #1 OR #2 | 33,011 |
| #4 | "Self Care"[MeSH Terms] | 67,799 |
| #5 | "self-care"[Title/Abstract] OR "foot-care"[Title/Abstract] OR "foot-care"[Title/Abstract] OR "foot self care"[Title/Abstract] OR "self-care"[Title/Abstract] OR "selfcare"[Title/Abstract] OR "self-management"[Title/Abstract] OR "self-management"[Title/Abstract] OR "preventive foot care"[Title/Abstract] OR ("self-care"[MeSH Terms] OR ("self"[All Fields] AND "care"[All Fields]) OR "self-care"[All Fields]) | 322,144 |
| #6 | #4 OR #5 | 322,144 |
| #7 | "health knowledge, attitudes, practice"[MeSH Terms] | 143,041 |
| #8 | "KAP"[Title/Abstract] OR "knowledge"[Title/Abstract] OR "attitude*"[Title/Abstract] OR "practice*"[Title/Abstract] OR "behavior*"[Title/Abstract] OR ("health knowledge, attitudes, practice"[MeSH Terms] OR ("health"[All Fields] AND "knowledge"[All Fields] AND "attitudes"[All Fields] AND "practice"[All Fields]) OR "practice attitudes health knowledge"[All Fields] OR "health knowledge attitudes practice"[All Fields]) | 3,786,989 |
| #9 | #7 OR #8 | 3,786,989 |
| #10 | "diabetes mellitus"[MeSH Terms] | 564,316 |
| #11 | "diabetes"[Title/Abstract] OR "mellitus"[Title/Abstract] OR "diabetes mellitus"[Title/Abstract] OR "diabet*"[Title/Abstract] | 918,546 |
| #12 | #10 OR #11 | 980,901 |
| #13 | #3 AND #6 AND #9 | 1,080 |
| Scopus | | |
| 3/2/2026 | | |
| #1 | ( TITLE-ABS-KEY ( diabetic foot ) OR TITLE-ABS-KEY ( diabetic feet ) OR TITLE-ABS-KEY ( diabetic foot ulcer* ) OR TITLE-ABS-KEY ( foot ulcer* ) ) | 46,346 |
| #2 | ( TITLE-ABS-KEY ( self care ) OR TITLE-ABS-KEY ( foot care ) OR TITLE-ABS-KEY ( foot-care ) OR TITLE-ABS-KEY ( foot self-care ) OR TITLE-ABS-KEY ( Self-Management ) ) | 448,915 |
| #3 | ( TITLE-ABS-KEY ( health knowledge ) OR TITLE-ABS-KEY ( health knowledge attitudes practice ) OR TITLE-ABS-KEY ( attitude to health ) | 5,170,665 |
| #4 | TITLE-ABS-KEY ( diabetes ) OR TITLE-ABS-KEY (diabetes mellitus) | 1,363,862 |
| #5 | #1 AND #2 AND #3 AND #4 | 906 |
| Embase | | |
| 3/2/2026 | | |
| #1 | ‘diabetic foot’/exp | 48,147 |
| #2 | ‘self care’/exp | 130,179 |
| #3 | 'attitude to health'/exp | 148,187 |
| #4 | 'diabetes mellitus'/exp | 1,560,707 |
| #5 | #1 and #2 and #3 and #4 | 120 |
| CINAHL Complete | | |
| 3/2/2026 | | |
| #1 | MH "Diabetic Foot+" OR XB ("diabetic foot" OR "diabetic feet" OR "diabetic foot ulcer*" OR DFU OR "foot ulcer*") | 14,428 |
| #2 | MH "Diabetes Mellitus+" OR TI (diabet* OR "diabetes mellitus" OR T2DM OR T1DM) | 283,033 |
| #3 | MH (self care) OR XB (self management) OR XB (self care) OR XB (foot care) OR XB (foot self-care) OR XB (self-care) | 81,693 |
| #4 | MH (health knowledge, attitudes, practice) OR XB (attitude to health) OR XB (kap) OR XB (knowledge) OR XB (attitude*) OR XB (practice*) | 884,373 |
| #5 | #1 AND #2 AND #3 AND #4 | 488 |
| Cochrane library | | |
| 3/2/2026 | | |
| #1 | MeSH descriptor: [Diabetic Foot] explode all trees | 1,681 |
| #2 | (diabetic foot OR diabetic feet OR diabetic foot ulcer OR DFU OR foot ulcer):ti,ab,kw | 5,495 |
| #3 | #1 OR #2 | 5,495 |
| #4 | MeSH descriptor: [Diabetes Mellitus] explode all trees | 48,118 |
| #5 | (Diabetes Mellitus OR diabet* OR T2DM OR T1DM):ti,ab,kw | 133,626 |
| #6 | #4 OR #5 | 134,036 |
| #7 | MeSH descriptor: [Self Care] explode all trees | 8,084 |
| #8 | ("foot care" OR foot-care OR foot self-care OR Self-Management):ti,ab,kw | 13,636 |
| #9 | #7 OR #8 | 19,667 |
| #10 | MeSH descriptor: [Health Knowledge, Attitudes, Practice] explode all trees | 8,824 |
| #11 | (KAP OR knowledge OR attitude* OR practice*):ti,ab,kw | 199,556 |
| #12 | #10 OR #11 | 199,556 |
| #13 | #3 AND #6 AND #9 AND #12 | 197 |
| Web of science | | |
| 3/2/2026 | | |
| #1 | TS=("diabetic foot" OR "diabetic feet" OR "diabetic foot ulcer*" OR DFU OR "foot ulcer*") | 14,753 |
| #2 | TS=(diabet* OR "diabetes mellitus" OR T2DM OR T1DM) | 648,904 |
| #3 | TS=("foot care" OR "foot-care" OR "foot self-care" OR "self care" OR selfcare OR "self-management") | 51,558 |
| #4 | TS=(KAP OR knowledge OR attitude* OR practice* OR health knowledge, attitude and practice ) | 2,393,333 |
| #5 | #1 AND #2 AND #3 AND #4 | 333 |
| CNKI | | |
| 3/2/2026 | | |
| #1 | SU=（糖尿病足 OR 糖尿病足溃疡） | 24,900 |
| #2 | SU=(足部护理 OR 足部自我护理 OR 足部保健 OR 足部护理行为 OR 自我管理 OR 自我护理) | 73,000 |
| #3 | SU=(知识 OR 态度 OR 行为 OR 知信行) | 594,980 |
| #4 | #1 AND #2 AND #3 | 890 |
| WanFang | | |
| 3/2/2026 | | |
| #1 | 主题:(糖尿病足) or 主题:(糖尿病足溃疡) | 37,522 |
| #2 | 主题:(足部护理) or 主题:(足部自我护理) or 主题:(足部保健) or 主题:(足部护理行为) or 主题:(自我管理 ) or 主题:(自我护理) | 215,465 |
| #3 | 主题:(知识) or 主题:(态度) or 主题:(行为) or主题: (知信行) | 11,179,694 |
| #4 | #1 AND #2 AND#3 | 1,177 |
| VIP Database | | |
| 3/2/2026 | | |
| #1 | 题名或关键词:(糖尿病足) or 题名或关键词:(糖尿病足溃疡) | 26,156 |
| #2 | 题名或关键词:(足部护理) or题名或关键词:(足部自我护理) or题名或关键词:(足部保健) or题名或关键词:(足部护理行为) or题名或关键词:(自我管理) or题名或关键词:(自我护理) | 24,922 |
| #3 | 题名或关键词:(知识) or题名或关键词:(态度) or题名或关键词:(行为) or题名或关键词: (知信行) | 1,707,497 |
| #4 | #1 AND #2 AND #3 | 210 |
| SinoMed | | |
| 3/2/2026 | | |
| #1 | ("糖尿病足溃疡"[常用字段:智能]) OR ("糖尿病足"[不加权:扩展]) | 31,178 |
| #2 | ("足部护理"[常用字段:智能] OR "足部自我护理"[常用字段:智能] OR "足部保健"[常用字段:智能] OR "足部护理行为"[常用字段:智能] OR "自我管理"[常用字段:智能] OR "自我护理"[常用字段:智能]) OR ("自我护理"[不加权:扩展]) | 147,947 |
| #3 | "知识"[常用字段:智能] OR "态度"[常用字段:智能] OR "行为"[常用字段:智能] AND "知信行"[常用字段:智能] | 1,964,975 |
| #4 | #1 AND #2 AND #3 | 395 |

**Supplementary Table S2** Methodological quality assessment of the included studies using the AHRQ checklist

| First author | Year | Q1 | Q2 | Q3 | Q4 | Q5 | Q6 | Q7 | Q8 | Q9 | Q10 | Q11 | Total score | Quality level |
| --- | --- | --- | --- | --- | --- | --- | --- | --- | --- | --- | --- | --- | --- | --- |
| Shi et al | 2010 | 1 | 1 | 1 | 0 | 0 | 1 | 0 | 0 | 0 | 1 | 0 | 6 | Moderate quality |
| Chen g et al | 2011 | 1 | 1 | 1 | 0 | 0 | 1 | 0 | 0 | 0 | 0 | 0 | 4 | Moderate quality |
| Huang et al | 2011 | 1 | 0 | 1 | 0 | 0 | 0 | 1 | 0 | 1 | 1 | 0 | 5 | Moderate quality |
| Zhang et al | 2013 | 1 | 1 | 1 | 1 | 0 | 0 | 1 | 0 | 0 | 1 | 0 | 6 | Moderate quality |
| Li et al | 2015 | 1 | 1 | 1 | 0 | 0 | 1 | 1 | 1 | 1 | 1 | 0 | 8 | High  quality |
| Wu et al | 2016 | 1 | 0 | 0 | 1 | 0 | 0 | 1 | 0 | 1 | 1 | 0 | 5 | Moderate quality |
| Fan | 2017 | 1 | 1 | 1 | 0 | 0 | 1 | 1 | 0 | 1 | 1 | 0 | 7 | Moderate quality |
| Qu et al | 2019 | 1 | 1 | 1 | 1 | 0 | 1 | 1 | 1 | 1 | 1 | 0 | 9 | High  quality |
| Xu | 2019 | 1 | 1 | 1 | 0 | 0 | 1 | 1 | 1 | 1 | 1 | 0 | 8 | High  quality |
| Liu | 2020 | 1 | 1 | 1 | 0 | 0 | 1 | 1 | 1 | 0 | 1 | 0 | 7 | Moderate quality |
| Xue | 2020 | 1 | 1 | 1 | 1 | 0 | 1 | 1 | 1 | 1 | 1 | 0 | 9 | High  quality |
| Yang et al | 2020 | 1 | 1 | 1 | 1 | 0 | 1 | 0 | 1 | 0 | 0 | 0 | 6 | Moderate quality |
| Long | 2021 | 1 | 1 | 1 | 0 | 0 | 1 | 1 | 1 | 1 | 1 | 0 | 8 | High  quality |
| Wang | 2021 | 1 | 1 | 1 | 0 | 0 | 1 | 1 | 1 | 0 | 1 | 0 | 7 | Moderate quality |
| Cai | 2021 | 1 | 1 | 1 | 0 | 0 | 0 | 1 | 1 | 0 | 1 | 0 | 6 | Moderate quality |
| Yang | 2021 | 1 | 1 | 1 | 0 | 0 | 1 | 1 | 1 | 0 | 1 | 0 | 7 | Moderate quality |
| Jia | 2022 | 1 | 1 | 1 | 1 | 0 | 1 | 1 | 1 | 0 | 1 | 0 | 8 | High  quality |
| Zhou | 2022 | 1 | 1 | 1 | 0 | 0 | 1 | 1 | 1 | 0 | 1 | 0 | 7 | Moderate quality |
| Xiong | 2023 | 1 | 1 | 1 | 0 | 0 | 1 | 0 | 1 | 1 | 0 | 0 | 6 | Moderate quality |
| Liu | 2023 | 1 | 1 | 1 | 0 | 0 | 1 | 1 | 1 | 1 | 1 | 0 | 8 | High  quality |
| Li | 2023 | 1 | 1 | 1 | 0 | 0 | 1 | 1 | 1 | 1 | 1 | 0 | 8 | High  quality |
| Cheng | 2023 | 1 | 1 | 1 | 1 | 0 | 1 | 0 | 1 | 0 | 0 | 0 | 6 | Moderate quality |
| Qi | 2025 | 1 | 1 | 1 | 1 | 0 | 1 | 1 | 0 | 1 | 1 | 0 | 8 | High  quality |
| Li | 2025 | 1 | 1 | 1 | 0 | 0 | 0 | 1 | 0 | 0 | 0 | 0 | 4 | Moderate quality |

**Note:** Q1, definition of information source; Q2, inclusion and exclusion criteria; Q3, time period for identifying patients; Q4, consecutive or population-based sampling; Q5, masking of evaluators; Q6, quality assurance assessment; Q7, explanation of patient exclusions; Q8, assessment or control of confounding; Q9, handling of missing data; Q10, response rate and completeness of data collection; Q11, follow-up or incomplete follow-up information. Each item was scored 1 for yes and 0 for no or unclear. Total scores of 0–3, 4–7, and 8–11 indicate low, moderate, and high quality, respectively.

**Supplementary Table S3** Leave-one-out sensitivity analysis of pooled proportions of knowledge, attitudes, and practices regarding diabetic foot care

| **Excluded study** | **Pooled proportion** | **Lower 95% CI** | **Upper 95% CI** |
| --- | --- | --- | --- |
| **Favorable attitude** |  |  |  |
| Shi_2010 | 0.709764 | 0.359034 | 0.957689 |
| Cai_2021 | 0.637042 | 0.276949 | 0.926098 |
| Yang_2021 | 0.756843 | 0.484895 | 0.948751 |
| Jia_2022 | 0.673493 | 0.302555 | 0.950163 |
| Qi_2025 | 0.546104 | 0.331429 | 0.752248 |
| **Unfavorable attitude** |  |  |  |
| Shi_2010 | 0.290236 | 0.042311 | 0.640966 |
| Cai_2021 | 0.362958 | 0.073902 | 0.723051 |
| Yang_2021 | 0.243157 | 0.051249 | 0.515105 |
| Jia_2022 | 0.326507 | 0.049837 | 0.697445 |
| Qi_2025 | 0.453896 | 0.247752 | 0.668571 |
| **Poor knowledge** |  |  |  |
| Cheng_2011 | 0.323583 | 0.175155 | 0.492665 |
| Huang_2011 | 0.297681 | 0.163253 | 0.452624 |
| Wu_2016 | 0.336647 | 0.187263 | 0.504769 |
| Fan_2017 | 0.287362 | 0.163203 | 0.430175 |
| Qu_2019 | 0.355795 | 0.212857 | 0.513080 |
| Xu_2019 | 0.327996 | 0.178938 | 0.497153 |
| Liu_2020 | 0.327016 | 0.177919 | 0.496373 |
| Xue_2020 | 0.285032 | 0.163679 | 0.424443 |
| Cai_2021 | 0.338510 | 0.189502 | 0.505869 |
| Long_2021 | 0.355024 | 0.211740 | 0.512842 |
| Wang_2021 | 0.327396 | 0.178481 | 0.496477 |
| Jia_2022 | 0.331525 | 0.181852 | 0.500846 |
| Liu_2022 | 0.323255 | 0.174812 | 0.492404 |
| Qi_2025 | 0.341606 | 0.192928 | 0.508040 |
| **Satisfactory knowledge** |  |  |  |
| Cheng_2011 | 0.427411 | 0.317797 | 0.540713 |
| Huang_2011 | 0.441473 | 0.333353 | 0.552445 |
| Wu_2016 | 0.409495 | 0.309520 | 0.513354 |
| Fan_2017 | 0.452518 | 0.350995 | 0.556053 |
| Qu_2019 | 0.417071 | 0.311103 | 0.527012 |
| Xu_2019 | 0.409729 | 0.309780 | 0.513551 |
| Liu_2020 | 0.439782 | 0.330868 | 0.551678 |
| Xue_2020 | 0.460940 | 0.368818 | 0.554411 |
| Cai_2021 | 0.416538 | 0.311460 | 0.525555 |
| Long_2021 | 0.431302 | 0.321247 | 0.544861 |
| Wang_2021 | 0.438826 | 0.329974 | 0.550709 |
| Jia_2022 | 0.423866 | 0.314852 | 0.536717 |
| Liu_2022 | 0.424314 | 0.315303 | 0.537138 |
| Qi_2025 | 0.435824 | 0.326024 | 0.548866 |
| **Good knowledge** |  |  |  |
| Cheng_2011 | 0.232252 | 0.139806 | 0.339675 |
| Wu_2016 | 0.239483 | 0.147991 | 0.344803 |
| Qu_2019 | 0.217394 | 0.129063 | 0.320963 |
| Liu_2020 | 0.216331 | 0.128878 | 0.318833 |
| Xue_2020 | 0.247217 | 0.159229 | 0.347163 |
| Cai_2021 | 0.229294 | 0.137291 | 0.336489 |
| Long_2021 | 0.205338 | 0.127526 | 0.295866 |
| Wang_2021 | 0.216969 | 0.129489 | 0.319455 |
| Jia_2022 | 0.227409 | 0.135233 | 0.335033 |
| Liu_2022 | 0.236638 | 0.144413 | 0.343243 |
| Qi_2025 | 0.208974 | 0.127309 | 0.304370 |
| **Poor practices** |  |  |  |
| Shi_2010 | 0.464683 | 0.366298 | 0.564458 |
| Huang_2011 | 0.421443 | 0.333264 | 0.512191 |
| Zhang_2013 | 0.460973 | 0.360200 | 0.563365 |
| Li_2015 | 0.442404 | 0.338898 | 0.548470 |
| Wu_2016 | 0.456961 | 0.354383 | 0.561394 |
| Xu_2019 | 0.443442 | 0.339963 | 0.549433 |
| Yang_2020 | 0.445283 | 0.341423 | 0.551587 |
| Cai_2021 | 0.465164 | 0.367148 | 0.564541 |
| Yang_2021 | 0.443037 | 0.339373 | 0.549239 |
| Zhou_2022 | 0.438822 | 0.336385 | 0.543930 |
| Jia_2022 | 0.429693 | 0.332534 | 0.529629 |
| Li_2022 | 0.437635 | 0.335784 | 0.542181 |
| Liu_2022 | 0.451539 | 0.347557 | 0.557678 |
| Xiong_2023 | 0.433510 | 0.333897 | 0.535878 |
| Cheng_2023 | 0.448862 | 0.344916 | 0.555089 |
| Li_2025 | 0.454972 | 0.351908 | 0.560000 |
| Qi_2025 | 0.465914 | 0.368222 | 0.564927 |
| **Satisfactory practices** |  |  |  |
| Shi_2010 | 0.446629 | 0.372351 | 0.522110 |
| Huang_2011 | 0.472744 | 0.408208 | 0.537735 |
| Zhang_2013 | 0.448171 | 0.373158 | 0.524375 |
| Li_2015 | 0.452067 | 0.376012 | 0.529251 |
| Wu_2016 | 0.441167 | 0.369781 | 0.513782 |
| Xu_2019 | 0.451329 | 0.375692 | 0.528100 |
| Yang_2020 | 0.452894 | 0.376966 | 0.529926 |
| Cai_2021 | 0.440850 | 0.369966 | 0.512952 |
| Yang_2021 | 0.457282 | 0.381370 | 0.534192 |
| Zhou_2022 | 0.465768 | 0.393589 | 0.538665 |
| Jia_2022 | 0.467287 | 0.396214 | 0.539025 |
| Li_2022 | 0.458197 | 0.382575 | 0.534787 |
| Liu_2022 | 0.450448 | 0.374646 | 0.527411 |
| Xiong_2023 | 0.463226 | 0.389646 | 0.537610 |
| Cheng_2023 | 0.449912 | 0.374546 | 0.526438 |
| Li_2025 | 0.448284 | 0.373355 | 0.524399 |
| Qi_2025 | 0.442392 | 0.370115 | 0.515900 |
| **GOOD practices** |  |  |  |
| Shi_2010 | 0.091256 | 0.058868 | 0.129642 |
| Zhang_2013 | 0.092027 | 0.058699 | 0.131666 |
| Wu_2016 | 0.103471 | 0.065888 | 0.148087 |
| Yang_2020 | 0.103260 | 0.065708 | 0.147858 |
| Cai_2021 | 0.096005 | 0.060093 | 0.138969 |
| Yang_2021 | 0.100492 | 0.062722 | 0.145654 |
| Zhou_2022 | 0.096022 | 0.059628 | 0.139652 |
| Jia_2022 | 0.107176 | 0.070806 | 0.149772 |
| Li_2022 | 0.108111 | 0.072532 | 0.149565 |
| Liu_2022 | 0.098279 | 0.061006 | 0.142949 |
| Xiong_2023 | 0.106014 | 0.069234 | 0.149285 |
| Cheng_2023 | 0.102382 | 0.064836 | 0.147061 |
| Li_2025 | 0.097885 | 0.061052 | 0.141983 |
| Qi_2025 | 0.093267 | 0.058786 | 0.134429 |

**Supplementary Figure S1** Leave-one-out sensitivity analysis for the pooled proportion of poor knowledge regarding diabetic foot care.

**Supplementary Figure S2** Leave-one-out sensitivity analysis for the pooled proportion of satisfactory knowledge regarding diabetic foot care.

**Supplementary Figure S3** Leave-one-out sensitivity analysis for the pooled proportion of good knowledge regarding diabetic foot care.

**Supplementary Figure S4** Leave-one-out sensitivity analysis for the pooled proportion of favorable attitude regarding diabetic foot care.

**Supplementary Figure S5** Leave-one-out sensitivity analysis for the pooled proportion of unfavorable attitude regarding diabetic foot care.

**Supplementary Figure S6** Leave-one-out sensitivity analysis for the pooled proportion of poor practice regarding diabetic foot care.

**Supplementary Figure S7** Leave-one-out sensitivity analysis for the pooled proportion of satisfactory practice regarding diabetic foot care.

**Supplementary Figure S8** Leave-one-out sensitivity analysis for the pooled proportion of good practice regarding diabetic foot care.

**Supplementary Figure S9** Trim-and-fill funnel plot for the pooled proportion of poor foot care practices.

**Supplementary Figure S10** Trim-and-fill funnel plot for the pooled proportion of satisfactory foot care practices.

**Supplementary Figure S11** Trim-and-fill funnel plot for the pooled proportion of good foot care practices.
